# A Deterministic Model for Understanding Nonlinear Viral Dynamics in Oysters

**DOI:** 10.1128/aem.02360-21

**Published:** 2022-03-29

**Authors:** Qubin Qin, Jian Shen, Kimberly S. Reece

**Affiliations:** a Virginia Institute of Marine Science, William & Mary, Gloucester Point, Virginia, USA; University of Tokyo

**Keywords:** in-host, virus-oyster interaction, OsHV-1, NoV, outbreaks, depuration, modeling, marine disease

## Abstract

Contamination of oysters with a variety of viruses is one key pathway to trigger outbreaks of massive oyster mortality as well as human illnesses, including gastroenteritis and hepatitis. Much effort has gone into examining the fate of viruses in contaminated oysters, yet the current state of knowledge of nonlinear virus-oyster interactions is not comprehensive because most studies have focused on a limited number of processes under a narrow range of experimental conditions. A framework is needed for describing the complex nonlinear virus-oyster interactions. Here, we introduce a mathematical model that includes key processes for viral dynamics in oysters, such as oyster filtration, viral replication, the antiviral immune response, apoptosis, autophagy, and selective accumulation. We evaluate the model performance for two groups of viruses, those that replicate in oysters (e.g., ostreid herpesvirus) and those that do not (e.g., norovirus), and show that this model simulates well the viral dynamics in oysters for both groups. The model analytically explains experimental findings and predicts how changes in different physiological processes and environmental conditions nonlinearly affect in-host viral dynamics, for example, that oysters at higher temperatures may be more resistant to infection by ostreid herpesvirus. It also provides new insight into food treatment for controlling outbreaks, for example, that depuration for reducing norovirus levels is more effective in environments where oyster filtration rates are higher. This study provides the foundation of a modeling framework to guide future experiments and numerical modeling for better prediction and management of outbreaks.

**IMPORTANCE** The fate of viruses in contaminated oysters has received a significant amount of attention in the fields of oyster aquaculture, food quality control, and public health. However, intensive studies through laboratory experiments and *in situ* observations are often conducted under a narrow range of experimental conditions and for a specific purpose in their respective fields. Given the complex interactions of various processes and nonlinear viral responses to changes in physiological and environmental conditions, a theoretical framework fully describing the viral dynamics in oysters is warranted to guide future studies from a top-down design. Here, we developed a process-based, in-host modeling framework that builds a bridge for better communications between different disciplines studying virus-oyster interactions.

## INTRODUCTION

The recognition of the importance of virus-oyster interactions is increasing, and the dynamics of viruses in oysters after infection have been recognized as a key linkage to the massive mortality of oysters (1) as well as human illnesses, including gastroenteritis and hepatitis outbreaks (2). Through their feeding process, shellfish can filter in viruses and become hosts to a variety of viruses that may be pathogenic to humans or to the oysters themselves.

A group of viruses (referred to as “group 1” here) that can infect oyster cells and replicate in oysters, such as ostreid herpesvirus 1 (OsHV-1), can have harmful impacts on oysters and, therefore, on oyster aquaculture industries. These viruses, particularly the OsHV-1 μvars, have caused massive mortalities of farmed Pacific oysters and severe economic losses in Europe, New Zealand, and Australia (1, 3, 4). The dynamics of viral concentration, oyster immune responses, and mortality are affected by the ontology of oysters and environmental conditions such as temperature and salinity (5–11). For example, de Lorgeril et al. (12) produced 15 biparental oyster families based on their resistance to OsHV-1 and found that susceptible families responded differently from resistant families after exposure. Virus in oyster tissues from the most susceptible family reached concentrations that were ∼100- to 1,000-fold higher than those in the most resistant family.

Other viruses do not replicate inside the shellfish and do not have adverse effects on the host (referred to as “group 2”). These include viruses such as norovirus (NoV), Tulane virus (TV), hepatitis A virus, and feline calicivirus. However, some viruses in this group, like human enteric viruses (e.g., hepatitis A virus and NoV), have been found to potentially impact human health through the consumption of shellfish like oysters and, therefore, threaten the seafood industry (13). This is because oysters, like many other shellfish species, are foods usually consumed either raw or undercooked and therefore serve as a vector for the transmission of these viruses. In recent decades, the accumulation of viruses in oysters has been linked to worldwide gastroenteritis or hepatitis outbreaks, which have caused great concern (2, 14). To reduce consumption risks, oysters grown in restricted areas such as those areas contaminated with human sewage are required to undergo postharvest treatment such as depuration or relaying, yet the efficacy of treatment to control outbreaks is thought to be limited (15).

There is evidence that oysters are not passive filters or ionic traps but rather that they have specific ligands that selectively accumulate viruses (16). Therefore, the efficacy of virus elimination through depuration can be affected. In fact, there are also data indicating that different virus types, and even those of the same type but in different genogroups, can have markedly different accumulation behaviors in oysters (17). Through depuration, some viruses such as feline calicivirus can be eliminated quickly from the oyster body, but others such as NoV can bind persistently to oyster tissues for a much longer period (18–20). In addition, both virus bioaccumulation and elimination are affected by environmental conditions such as water temperature and salinity (19, 21, 22). Thus, a comprehensive understanding of the dynamics of the different types of viruses in oysters is required to accurately predict bioaccumulation and depuration.

Thorough knowledge of the interaction between viruses and oysters is needed to support management decisions (15), which has been partially accomplished by advances made through numerous laboratory experiments and field observations. The understanding of oyster-virus interactions involves studies in multiple disciplines like virology, marine science, aquaculture, and food science. Nevertheless, studies in each discipline have their own focus, and gaps in knowledge exist between disciplines. For example, the filtration behavior of oysters is recognized as the major means of virus uptake (filter-in) and an important way for oysters to clear virus particles (filter-out or viral shedding), and marine biologists know well that different environmental conditions can greatly alter the filtration rate (23, 24); however, the effects of filtration on the efficacy of depuration are largely overlooked in virology or aquaculture and food sciences. The possible contribution of different filtration rates is seldom discussed as a factor when explaining the variations in depuration efficacies found among laboratory experiments, and experiments directly evaluating the effects of different filtration rates on depuration have never been conducted. Thus, to fill knowledge gaps and to guide future studies, a framework that describes the dominant processes controlling viral dynamics in oysters and the influence of various environmental and physiological factors is warranted.

Although the importance of marine viruses in the ecosystem is recognized, there are few mathematical models describing oyster-virus interactions within oysters. Many existing models, including those examining virus-host interactions in marine systems, are focused on viral transmission between host individuals and host population dynamics, and most of them are developed for studying the population dynamics of organisms other than shellfish, such as phytoplankton and bacterioplankton (25). In these models, the processes regulating viral dynamics within the host are largely simplified (25). On the other hand, those models that are used to study interactions between marine shellfish and disease are rarely developed for viruses (26, 27). Bidegain et al. (28) theoretically provide a series of marine infective disease models that may be used for interactions between oysters and infectious viruses such as OsHV-1; however, the models are transmission models for investigating host population dynamics and viral transmission between hosts, and they do not consider in-host interactions. Polo et al. (29, 30) proposed a simple mathematical model for norovirus, considering filtration for viral depuration based on experimental data, but the model does not simulate the process of viral bioaccumulation.

Here, we propose a new modeling framework that includes all essential processes when oysters are contaminated by viruses. The model is for in-host viral dynamics in one oyster and investigates virus infection between targeted tissue cells in that oyster.

## RESULTS

### Modeling in-host viral dynamics.

In-host dynamics are governed by three coupled equations, which are (i) the uninfected target cell, *T* (grams per oyster); (ii) the infected cell, *I* (grams per oyster); and (iii) the virus concentration in oyster tissues, *V* (copies per gram), respectively:

(1) $$\frac{dT}{dt}=-\beta TV-\delta_{T}T+s_{T}$$

(2) $$\frac{dI}{dt}=\beta TV-\delta I$$

(3) $$\frac{dV}{dt}=\frac{pI}{m}-\frac{a}{m}\beta TV+\frac{\varepsilon_{i}}{m}fV_{l}-\frac{\varepsilon_{o}}{\sigma }fV-cV$$

with an initial condition (*T*_0_, *I*_0_, *V*_0_). The definition of each parameter is listed in Table A1 in the appendix. *t* (days) denotes time. *m* (grams per oyster) is the weight of the total target cells in one oyster if the oyster is not infected. β (grams per copy per day) is the infection rate. Note that β is considered on the scale of host cells within an oyster, which reflects the rate of entry of the virus into the host cells, a concept that differs from the concept of virus infectivity or virulence in studies focusing on virus transmission between host individuals. δ*_T_* (per day) and δ (per day) are the death rates of uninfected and infected target cells, respectively, and it is naturally assumed that for oyster-pathogenic viruses, δ is less than or equal to δ*_T_*. *s_T_* (grams per oyster per day) is the production rate of new uninfected target cells. New uninfected cells can be generated (i) by the replacement of old cells that have been eliminated through natural death (δ*_T_T*), (ii) by increasing cell numbers through oyster growth, and (iii) as a host response to infection that leads to an increase in cells, such as hemocyte infiltration in response to pathogens or at wound sites in oysters. *p* (copies per gram per day) is the rate of virus replication in infected cells. Because virus replication requires the infection of target cells, and this process results in the loss of those viruses from the oyster tissues through either the absorption of the viral particle into the cell or the injection of viral components such as nucleic acids into the cell to induce the production of new virus (31), a loss term is included in the equation −(*a*/*m*)β*TV*; *a* (copies per gram) describes how many copies of viruses are needed to infect 1 g of target cells. *V_l_* (copies per cubic meter) is the virus concentration in the surrounding water, *f* (cubic meter per day per oyster) is the filtration rate, and *c* (per day) is the in-host virus clearance/elimination rate. σ (cubic meters per oyster) is the volume of the individual oyster. ε*_i_* is the bioaccumulation fraction in the filter-in process; ε*_o_* is the fraction of virus that cannot be retained in oyster tissues during filter-out/shedding processes, referred to as the shedding fraction; and the two parameters depend on both selective accumulation and in-host physical trapping. An ε*_i_* value of 1 means that all particles that are filtered into the oyster bind the tissues, while an ε*_i_* value of 0 means that no filtered-in virus can accumulate in oyster tissues. An ε*_o_* value of 0 means that the process of filtration cannot remove virus particles from the oyster body, while an ε*_o_* value of 1 means that virus particles in oyster tissues are freely filtered out. Clearly, ε*_i_* and ε*_o_* show a negative relationship; i.e., a higher ε*_i_* corresponds to a lower ε*_o_*. In equation 3, the term (*pI*/*m*) − (*a*/*m*)β*TV* describes the in-host production of copies of the virus by infected cells, and the terms (ε*_i_*/*m*)*fV_l_* − (ε*_o_*/σ)*fV* and −*cV* describe net virus uptake through filtration (i.e., filter-in minus filter-out) and in-host clearance, respectively.

For group 2 viruses (i.e., viruses that do not replicate), β = *p* = 0 so that equation 3 becomes

(4) $$\frac{dV}{dt}=\frac{\varepsilon_{i}}{m}fV_{l}-\left(\frac{\varepsilon_{o}}{\sigma }f+c\right)V$$

All parameters in the in-host model can be time dependent to resemble the real viral dynamics in oysters in natural systems that are affected by various environmental conditions and ecophysiologies. Note that *V_l_* is the variable connecting to the aquatic system, and it can be linked to a dynamic virus transport model in the water.

In natural waters, oysters are usually cultured for a relatively long time, and it is sometimes necessary to study the long-term accumulation of viruses in oysters. With long-term exposure to a virus, the concentration in oysters is expected to be varying around equilibrium values. Long-term viral dynamics for either group 1 or 2 viruses can be examined by the nontrivial steady-state solution for *V* that reads

(5) $$V^{*}=\frac{q+\frac{\varepsilon_{i}}{m}fV_{l}}{\frac{\varepsilon_{o}}{\sigma }f+c}$$

where *q* = (*p*/*m*/δ − *a*/*m*)(*s_T_* − δ_T_T*), which represents the steady-state net in-host viral production, in addition to the filter-in, (ε_i_/*m*)*fV_l_*, from the water. All parameters in equation 5 are long-term equilibrium values. For group 1 viruses, if *V_l_* is zero, there is another trivial equilibrium (V* = 0), indicating that the virus can be removed completely from the oyster tissues. Whether *V* approaches trivial or nontrivial equilibrium depends on the particular conditions (see Section S1 in the supplemental material). For group 2 viruses, *q* is zero, and V* is proportional to *V_l_*. If *V_l_* becomes zero, V* approaches zero.

### Physiological meaning of model parameters and their variations.

Many abiotic and biotic factors impact the various processes regulating viral accumulation and dynamics in oysters, including virus type; oyster factors such as size, age, species, the selective accumulation of some viruses, and host antiviral responses; and environmental factors such as temperature and salinity. These factors and their impacts are accounted for in the model through changes in parameter values.

For both groups of viruses, the concentration in the water, *V_l_*, is determined by the fate and transport of the virus following discharge into the system. This means that the viral concentration in oyster tissues is determined by the overall concentration of the virus in the water rather than the loading pattern (chronic or accidental exposure to virus), which agrees with previously reported experimental results (32) and indicates that this conclusion also holds for different virus types. For bioaccumulation experiments that have different loading patterns (e.g., an oyster either is exposed to the virus at the beginning of an experiment at a specific time or is repeatedly exposed during the course of the experiment), *V_l_*, however, needs to be carefully evaluated. Either a constant value or time-variable values are used because *V_l_* may differ from the initial level through the length of the oyster simulation period.

The filtration rate, *f*, differs depending on the oyster species, size, and various environmental factors. For a specific oyster species, *f* can be expressed explicitly as it is a function of several environmental factors such as temperature, salinity, the dry weight of the oyster, and total suspended solids (TSS) if observational data are available. The function can vary among oyster species (23, 24, 33). For example, Crassostrea virginica reaches its highest filtration rate at around 27°C (24), while Crassostrea gigas reaches its highest rate at a lower temperature of around 19°C (34).

Viruses use host metabolic machinery for replication, and their replication rate, *p*, is dependent on the structure of the virus, the length of the latent period, whether it goes through a lytic or a lysogenic cycle, the growth conditions, and the physiology of oyster cells (35). *p* is also affected by the antiviral responses of oysters. Oysters have an innate, but not an adaptive, immune system (36), and the antiviral defense in oysters has been investigated extensively in recent years (37). The oyster’s type I interferon (IFN)-like pathway is considered one of the major strategies of innate immunity, along with other pathways such as autophagy (38) and apoptosis (39). These pathways result in the suppression of viral replication (decreased *p*, e.g., through an IFN-like response) and an increase in clearance (increased *c*, e.g., through phagocytosis).

It has been suggested that apoptosis is an important defense mechanism that the oyster uses against OsHV-1 by limiting viral spread and eliminating infected cells (39). Inhibition of apoptosis, regulated by both oysters and viruses, has been observed in both juvenile and adult oysters in OsHV-1 infection experiments (40, 41). Besides apoptosis, autophagy may also play a role in eliminating infected cells (38), and the effects of the two host processes on viral dynamics are expressed by the death rate of infected cells, δ, in the in-host model. The long-term net production rate of the virus is determined by the ratio of *p* to δ in *q* in equation 5. This model indicates that the inhibition of apoptosis (reduced δ) can greatly facilitate viral production in oysters.

Oyster antiviral immune responses vary with ontogeny, physiology, and history of infection (37, 42). Oysters can acclimate to changes in environmental conditions such as temperature and salinity, and environmental conditions are key factors modulating oyster immune defense. The expression levels of more than half of the immune genes found in the C. gigas genome are responsive to changes in environmental conditions (43). For example, a vigorous antiviral response in juvenile *C. gigas* is observed at 22°C compared to an inhibited response at 12°C, which indicates that the higher temperature within the suitable range for *C. gigas* facilitates antiviral defense (7). The expression of proapoptotic and autophagy genes also increases with higher temperatures (44). On the other hand, extreme conditions may impair the immune response. Hemocyte activity in *C. gigas* shows an increasing trend with temperature but a significant decrease at temperatures above 50°C (45). More investigation into the immune responses under different environmental conditions has been recommended (46) given that there is relatively little information in the literature on this topic.

Many group 1 oyster viruses can enter hemocyte cells and be retained for significantly longer periods, increasing the time for filtering out. At the same time, hemocytes can also inactivate these viruses. Therefore, this competition can either increase or decrease the in-host elimination rate, *c*. *c* is also impacted largely by other clearance strategies, such as extracellular inactivation through the digestive process (47).

Infectivity is a key parameter in determining the course of the host-virus interaction inside the oyster. Higher infectivity can reduce the time required for the virus to reach the peak level in oysters. The infectivity rate (β) depends on the virus type, oyster species, and mechanisms of entry of the virus into host cells. The envelopes of some virus types can promote transmission from one host cell to another (48). In addition, infectivity is affected by environmental conditions such as temperature and salinity (49). It has been suggested that high temperature may decrease the infectivity of OsHV-1, an enveloped virus, by altering the membrane composition, limiting the entry of the virus into host cells (44). Low salinity (e.g., 10 ppt) is also suggested to significantly reduce the infectivity of OsHV-1 (10).

The selective accumulation of virus (expressed by the bioaccumulation fraction, ε*_i_*, and the shedding fraction, ε*_o_*) varies among virus species (18) and can even differ among virus genogroups. Studies have found that a human blood group antigen A (HBGA)-like carbohydrate ligand is found in the oyster digestive tract, allowing noroviruses, specifically NoVs in genogroup I (GI), to preferentially and most strongly bind to the oyster gut over other tissues, while those NoVs in GII bind to the gut but also to other oyster tissues, including the gills and mantle (16, 17, 50). In addition, GI NoVs are more tightly bound to oyster tissues overall than GII NoVs and exhibit larger seasonal variability. Many have found that the accumulation of NoV GI in oysters is higher in winter (17, 50–53), which may be because the HBGA-like carbohydrate ligands in the oyster digestive tract are more highly expressed during the colder months of the year (17).

### Model simulations with example case studies.

**FIG 1:**
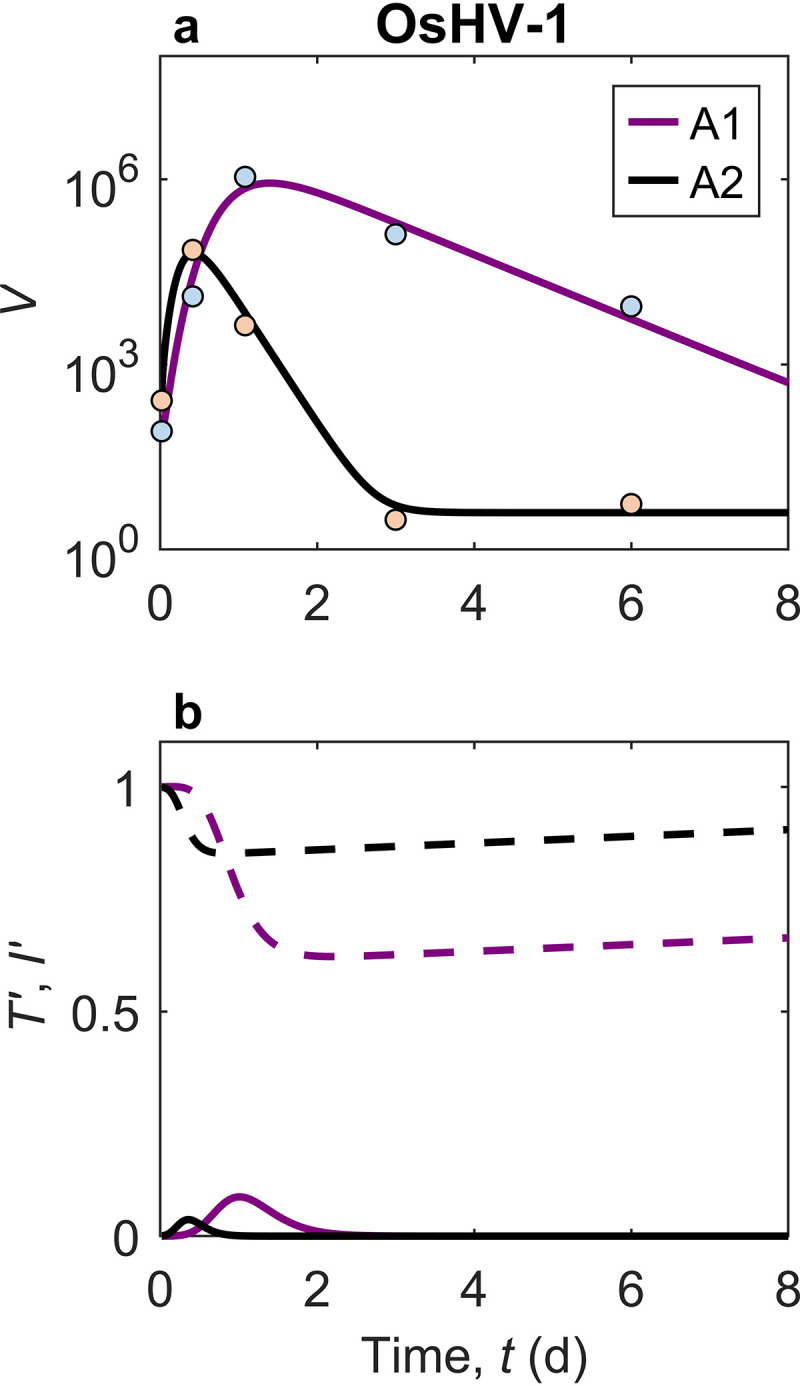
Viral dynamics in oysters for ostreid herpesvirus (OsHV-1) simulated by the in-host model. (a) Dynamics of *V* (viral DNA copies per nanogram of oyster DNA) in Pacific oysters, fitting the laboratory data reported previously by Segarra et al. (39) for two oyster families, A1 (*r*^2^ = 0.97; *P* = 1.7 × 10^−3^) and A2 (*r*^2^ > 0.99; *P* = 4.9 × 10^−5^). (b) Dynamics of normalized uninfected target cells, *T*′ (dashed lines), and normalized infected target cells, *I*′ (solid lines), for the two oyster families, respectively. Values used in the model are listed in Table 1.

**FIG 2:**
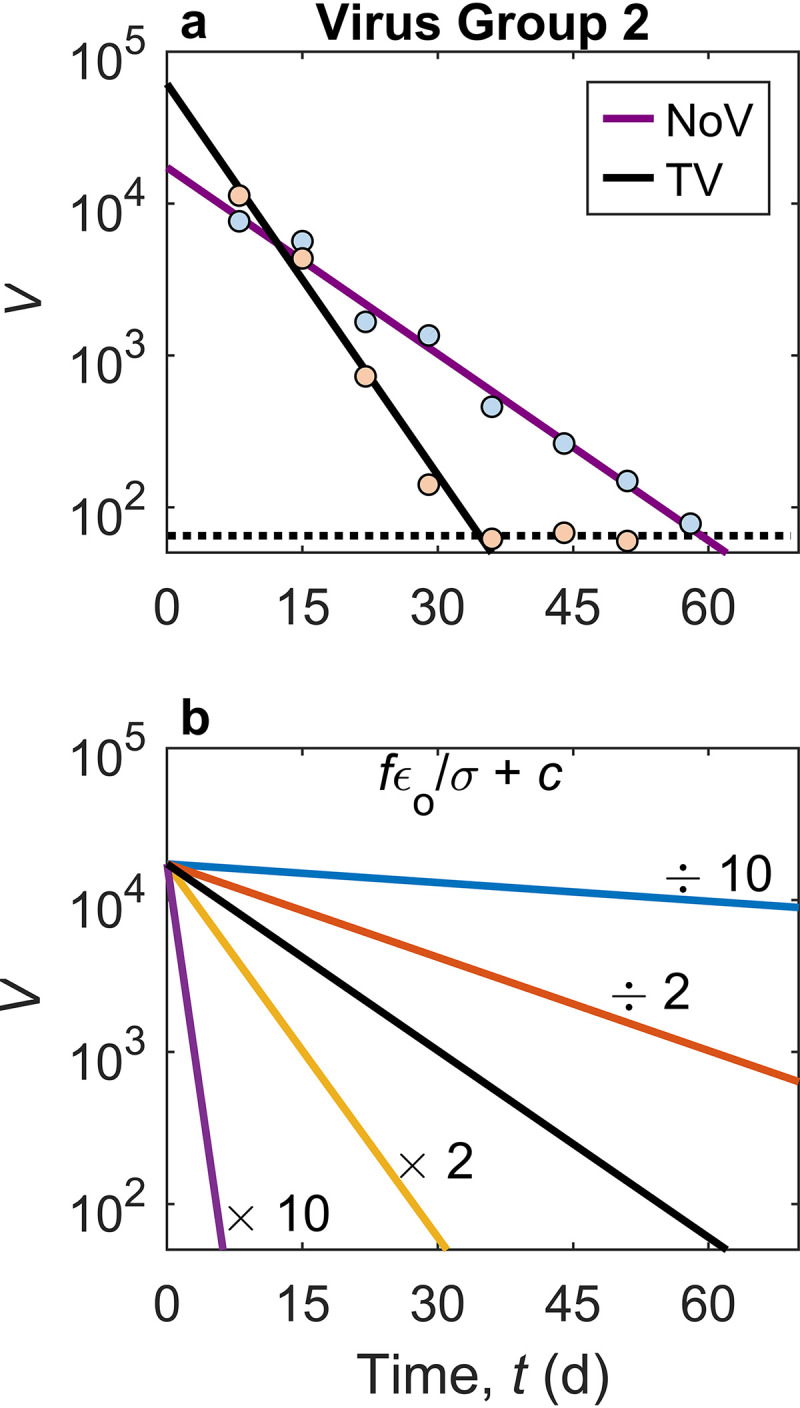
Viral dynamics in oysters for norovirus (NoV) and Tulane virus (TV) simulated by the in-host model. (a) Dynamics of *V* in digestive tissue (DT) of Pacific oysters (viral RNA copies per gram of DT) for NoV (*r*^2^ = 0.98; *P* < 10^−6^) and TV (*r*^2^ = 0.98; *P* < 10^−3^), fitting the laboratory data reported previously by Drouaz et al. (54). The dotted line denotes the limit of quantification in the experiments. (b) Sensitivity of viral dynamics of group 2 viruses in oysters to the removal rate, (ε*_o_*/σ)*f* + *c*, in the model in the depuration process. The base uses the parameter set for simulating the viral dynamics of NoV in oysters (denoted by the black line).

The modeling framework can be used for theoretical analysis and predictions of real viral case studies or laboratory experimental results. Fitting the model against experimental data provides the range of parameter values in the model and allows numerical examination of the effects of each process by altering the values of related parameters. Data from two laboratory experiments were fitted using the normalized model (see equations 25 to 27) for viruses in groups 1 and 2 (Fig. 1 and 2, respectively), using the values for the parameters listed in Table 1. The model predicts the viral dynamics in oyster tissues after infection with group 1 viruses (Fig. 1): viral levels increase largely during the acute phase and decrease either slightly to a stable level or more substantially to a low level. This process is predicted by the model and agrees well with observations from laboratory experiments, which had not been conducted until recently (12, 39). For example, as a representative of group 1 viruses, OsHV-1 replicates and accumulates rapidly in oyster tissues during the acute phase, as described previously by Segarra et al. (39). The dynamics, however, have been shown to differ in viral levels between oyster families A1 and A2, and the variation in progression is well described by the model (Fig. 1a). Family A2 shows an earlier acute phase but a lower peak level than family A1 and drops to a lower stable level, while virus levels in family A1 drop steadily after the later peak. Figure 1b shows the variations in normalized uninfected target cells, *T*′, and normalized infected target cells, *I*′.

**TABLE 1:** List of parameters required in the transformed mathematical model^*a*^ and their values used in the examples resembling culture experiments reported previously^*b*^^,^^*c*^

Parameter	Description	Value			
		Virus group 1		Virus group 2	
		OsHV-1, A1 family	OsHV-1, A2 family	NoV	TV
$\text{θ}p_{0}^{\prime }$ (viral DNA copies/ng of oyster DNA/day)	Initial rate of virus replication by infected cells	3.75 × 10^7^	3.75 × 10^7^		
*k_p_* (day^−1^)	Decay rate for *p*	0.5	3.5		
β_0_ (ng of oyster DNA/viral DNA copy/day)	Initial infection rate	1.8 × 10^−5^	5.0 × 10^−5^		
*k*_β_ (day^−1^)	Decay rate for β	3.0	5.5		
δ (day^−1^)	Death rate of infected target cells	4.8	10		
s′_T_net (day^−1^)	Net production rate of uninfected target cells	7.5 × 10^−3^	7.5 × 10^−3^		
θε*_i_fV_i_*	Filter-in	18 viral DNA copies/ng of oyster DNA/day	18 viral DNA copies/ng of oyster DNA/day	0 viral RNA copies/g of DT/day	0 viral RNA copies/g of DT/day
$\frac{{\text{ε}}_{o}f}{\text{σ}}+c$ (day^−1^)	Removal rate	1.2	4.5	9.43 × 10^−2^	1.98 × 10^−1^
$I_{0}^{\prime }$	Normalized initial infected target cells	1 × 10^−5^	1 × 10^−3^		
*V*_0_	Initial virus concn in oysters	8.32 × 10^1^ viral DNA copies/ng of oyster DNA	2.62 × 10^2^ viral DNA copies/ng of oyster DNA	1.73 × 10^4^ viral RNA copies/g of DT	6.13 × 10^4^ viral RNA copies/g of DT

a See equations 25 to 27.

b Experiments reported previously by Segarra et al. (39) and Drouaz et al. (54).

c See Fig. 1 and 2. OsHV-1, ostreid herpesvirus; NoV, norovirus; TV, Tulane virus; DT, digestive tissue. Note that $T_{0}^{\prime }=1-I_{0}^{\prime }$ was assumed for the examples, and the values of $I_{0}^{\prime }$ and *V*_0_ listed here for OsHV-1 were values at 0.5 h used in the model, in order to be consistent with the laboratory data reported by Segarra et al. (39). The first virus concentrations after infection were reported at 0.5 h. The values of parameters except for *V*_0_ are calibrated.

Using NoV and TV as two representatives of group 2 viruses, the model describes the depuration process as observed in the laboratory well (Fig. 2a). The calculated removal rate of NoV in *Crassostrea gigas* is 9.43 × 10^−2^ day^−1^ for the experiment described previously by Drouaz et al. (54) (Table 1). This value is close to that calculated from other depuration experiments conducted at similar temperatures (15). The removal rate of TV is 1.98 × 10^−1^ day^−1^, which is more than double that of NoV under similar conditions. The bioaccumulation efficiency was also estimated based on the first hour of the bioaccumulation experiments described by Drouaz et al. (54), and the bioaccumulation fraction, ε*_i_*, for NoV (ranging from 1.96 × 10^−4^ to 9.83 × 10^−4^) was estimated to be about 60-fold higher than the ε*_i_* for TV (ranging from 3.23 × 10^−6^ to 1.61 × 10^−5^).

### Parameter-induced changes in viral dynamics.

**FIG 3:**
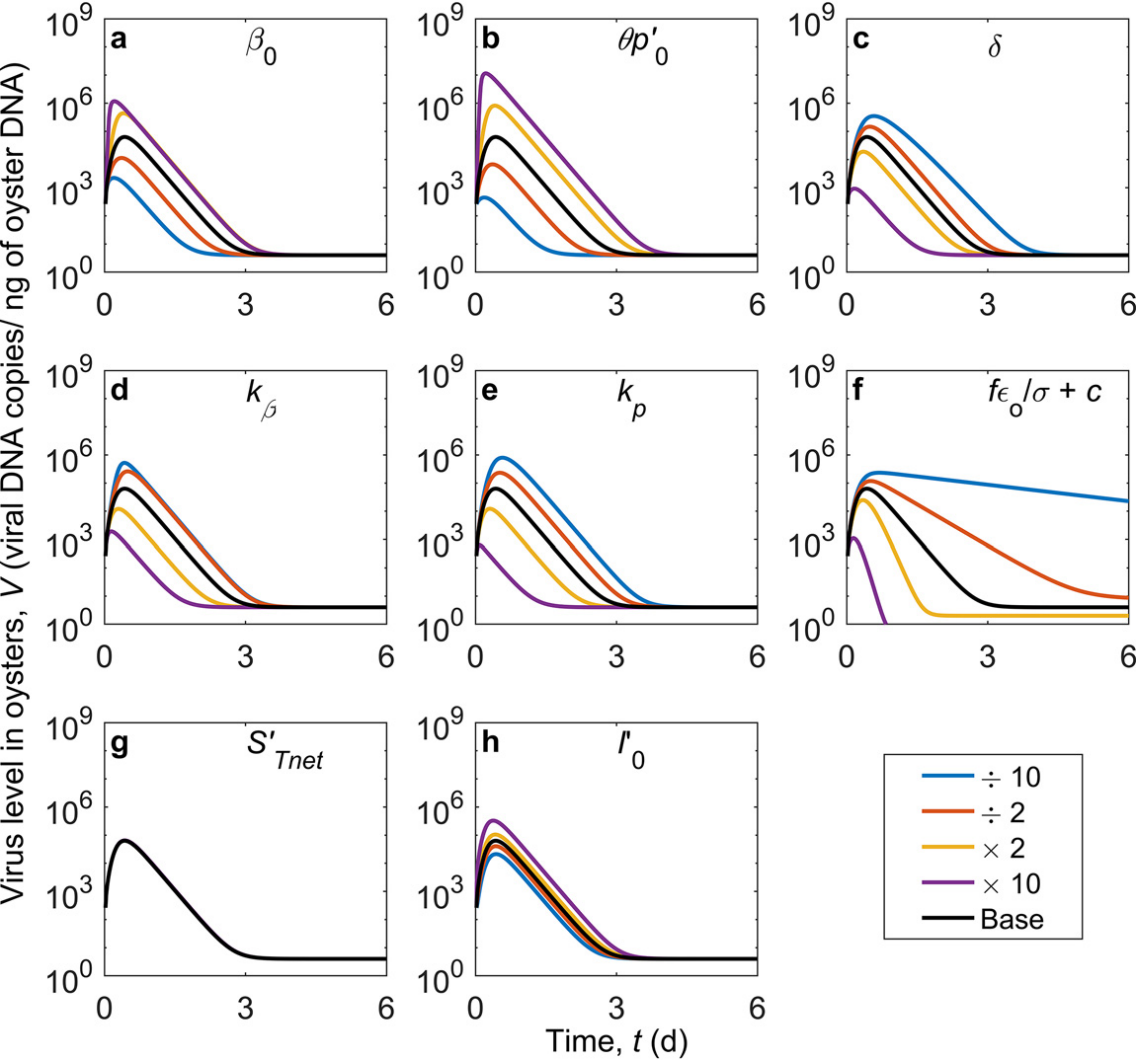
Sensitivity of viral dynamics of group 1 viruses in oysters to parameters in the model (Table 1). The base uses the parameter set for simulating viral dynamics of OsHV-1 in oysters (A2 family) (denoted by the black lines).

The model provides a means to examine changes in viral dynamics with variation in parameter values that are associated with different processes. For group 1 viruses, the response to changes in parameter values that family A2 had to OsHV-1 infection is an example (Fig. 3 and Table 2). Although the response of the virus concentration in oysters, *V*, to changes in parameter values is not linear, an increase in the infection rate, β (increased β_0_ or decreased *k*_β_ in the normalized model in equations 25 to 27), or the viral replication rate, *p* (increased $\theta p_{0}^{\prime }$ or decreased *k_p_* in the normalized model), will generally shorten the time required to reach the peak and increase the peak level of the virus in oyster tissues (Fig. 3a, b, d, and e). An increase in the death rate of infected cells, δ, or the removal rate, (ε*_o_*/σ)*f* + *c*, will decrease *V* (Fig. 3c and f). The removal rate determines the decrease rate of the virus level after the peak. A change in the net production rate of new uninfected target cells, s′_T_net, does not significantly alter *V* (Fig. 3g), while an increase in the initial fraction of infected cells, $I_{0}^{\prime }$, will slightly increase *V* and shorten the time to the peak (Fig. 3h). For the depuration of group 2 viruses, an increase in the removal rate will facilitate the decrease of virus in oyster tissues (Fig. 2b), which corresponds to an increase in any of the three parameters filtration rate, *f*; in-host elimination rate, *c*; and shedding fraction, ε*_o_*.

**TABLE 2:** Changes of viral dynamics of group 1 viruses in oysters in each sensitivity case, such as the ratio of the peak virus concentration in each case to that in the base scenario and the shift in the time when the virus concentration reaches the peak^*a*^

Case	β_0_	$\text{θ}p_{0}^{\prime }$	δ	*k*_β_	*k_p_*	$\frac{\varepsilon_{o}f}{\sigma }+c$	s′_T_net	$I_{0}^{\prime }$
	Ratio of peak virus concn							
÷10	0.035	0.007	5.533	8.273	12.593	3.686	0.997	0.335
÷2	0.180	0.107	2.316	4.114	3.675	1.868	0.998	0.640
×2	6.988	12.982	0.301	0.191	0.193	0.391	1.003	1.660
×10	18.658	182.046	0.015	0.030	0.010	0.018	1.028	5.186

	Shift in time (days) when virus concn reaches the peak							
÷10	−0.229	−0.242	0.149	−0.004	0.143	0.267	0.000	0.008
÷2	−0.067	−0.067	0.063	0.063	0.088	0.082	0.000	0.004
×2	−0.027	−0.018	−0.075	−0.132	−0.124	−0.084	0.000	−0.008
×10	−0.219	−0.212	−0.264	−0.285	−0.344	−0.286	0.002	−0.052

a Note that the base scenario uses the parameter set of OsHV-1 in oysters (A2 family) in Table 1, and the peak virus concentration and the time to reach the peak in the base scenario are about 6.335 × 10^4^ viral DNA copies/ng of oyster DNA and 0.428 days, respectively.

For long-term equilibrium, equation 5 suggests that the equilibrium value, V*, always shows positive relationships with the net in-host viral production, *q*; bioaccumulation fraction, ε*_i_*; and virus concentration in the surrounding water, *V_l_*. On the other hand, the in-host elimination rate, *c*, and the shedding fraction, ε*_o_*, demonstrate a negative relationship with viral accumulation. The response of V* to changes in the filtration rate, *f*, varies. For group 2 viruses (*q *= 0), V* always increases with *f*, but V* may either increase or decrease with *f* for group 1 viruses. Also, an increased filter-in value, (ε*_i_*/*m*)*fV_l_*, will shorten the time to reach bioaccumulation equilibrium for group 2 viruses and contribute more to the dynamics of group 1 viruses than with the net in-host viral production denoted by *q*.

## DISCUSSION

### Virus group 1: infection and long-term equilibrium.

Oysters of all ages and sizes can be affected by OsHV-1, particularly by the μvar strains. It has been suggested that larger and older oysters can be more resistant to OsHV-1 infection and that size may have a greater influence than age (55). Generally, adult oysters are found to be more resistant to OsHV-1 infection than juveniles, possibly due to the maturation of the immune system, while the higher growth rate of juvenile oysters may facilitate the in-host replication of the virus (9). In the model framework, this corresponds to a lower viral replication rate, *p*, or infection rate, β; a higher in-host elimination rate, *c*; and a lower production rate of new uninfected target cells, *s_T_*, in adult oysters than in juvenile oysters. Also, the resistance of oysters to ostreid herpesvirus disease varies among different oyster species and stocks and differs with exposure to OsHV-1 strains, including among the different OsHV-1 μvars (56–58).

de Lorgeril et al. (12) produced 15 biparental oyster families based on their resistance to an OsHV-1 μvar variant, and they found that the response of the susceptible families was significantly different from that of the resistant families after exposure to the virus. When the virus concentration approached equilibrium, one susceptible family reached a virus concentration that was about 100- to 1,000-fold higher than the concentration in one of the resistant families. A differential immune response in the two families is suggested to be the main reason for the difference in viral loads. The weak response in the susceptible family indicates a high viral replication rate, *p*, but a low in-host elimination rate, *c*, and the overexpression of proteins that inhibit apoptosis also facilitates viral replication (reduced death rate of infected cells, δ). This is demonstrated by the model parameters of the two families A1 and A2 in the case study (Table 1). In addition, because of possibly higher net in-host viral production, *q*, the susceptible family will have higher virus levels than the resistant family over the long term in surviving oysters, as suggested by equation 5.

Long-term equilibrium is influenced by environmental conditions. For example, salinity can affect the bioaccumulation of marine viruses. OsHV-1 accumulated to similar levels in oysters cultured at salinities of 15, 25, and 35 ppt but to a much lower level at a salinity of 10 ppt (10). This suggests that higher salinities result in higher bioaccumulation. While salinity may affect host metabolism and the infectivity of OsHV-1 (11), the in-host model suggests that the salinity effect on bioaccumulation may be also through the regulation of the oyster filtration rate. This is consistent with the observations that the filtration rate measured at a salinity of 10 ppt is significantly lower than that at higher salinities (10). With a higher filtration rate and with relatively little change in other parameters, the in-host model shows a higher equilibrium value.

Temperature also affects the in-host dynamics of OsHV-1 in oysters. OsHV-1 infections appear to be more frequent during summer (59–62). The transmission of OsHV-1 occurred and infections were established at temperatures below 16°C; however, this did not result in massive mortality (8). For quantitatively examining the impacts of temperature on viral dynamics in oysters, we conducted a numerical modeling experiment to examine the impacts of temperature on OsHV-1 infection in oysters, based on the parameter values specified in Table 1 for *C. gigas* family A2 at 22°C. In this particular experiment, it may be reasonably assumed that the shedding fraction, ε*_o_*, for OsHV-1 during filter-out/shedding processes is close to ε*_o_* for group 2 viruses (e.g., NoV and TV), that is, on the order of 0.1 day^−1^ (Table 1), and therefore, the two components of the removal rate, *c* and ε*_o_f*/σ, are assumed to be 4.2 day^−1^ and 0.3 day^−1^ at 22°C, respectively. The response of the filtration rate (*f*) for *C. gigas* to temperature follows the measurement reported previously by Bougrier et al. (34), and the effect of temperature on the metabolic rates of oysters is assumed to be equal to the reported effect of temperature on the oxygen consumption rate of oysters (34), which leads to a *Q*_10_ temperature coefficient of 1.5, where *Q*_10_ describes the factor by which the rate changes with a temperature increase of 10°C. Higher water temperatures are thought to promote more rapid viral replication and higher mortality rates of infected oysters (63), and mathematically, this indicates that a higher temperature may correspond to a higher initial rate of virus replication ($\theta p_{0}^{\prime }$) in the transformed model. On the other hand, at higher temperatures, the antiviral immune defense in oysters is also enhanced (6, 44), which may inhibit viral increases. For example, in terms of causing mortality, the upper thermal limits are suggested to be 22°C to 25°C in the Thau Lagoon, France (5). Generally, the observed responses of immune activities to temperature indicate that increasing temperature increases the decay rates for the viral replication rate (*k_p_*) and for the infection rate (*k*_β_) and also the in-host elimination rate (*c*) in the model. Particularly, it is suggested that higher temperatures may reduce the viral levels by limiting OsHV-1 entry into oyster cells (44), which corresponds to a decrease in the initial infection rate (β_0_). The death rate of infected target cells (δ) is highly controlled by apoptosis. It is regulated by both pro- and antiapoptotic effects in oysters, which in turn may be influenced by the presence of the virus itself. The expression of both pro- and antiapoptotic genes is upregulated with an increase in temperature (44), and therefore, the net effect and relationship to temperature remain unclear. To consider this uncertainty, the model experiment contains three scenarios for OsHV-1 infection. Scenario 1 assumes that the net apoptotic effect remains relatively constant (i.e., constant δ) over the range of temperatures, scenario 2 assumes that a lower temperature corresponds to a greater proapoptotic effect and that δ decreases with the increase in temperature, and scenario 3 assumes that a lower temperature corresponds to a greater antiapoptotic effect and that δ increases with temperature.

**FIG 4:**
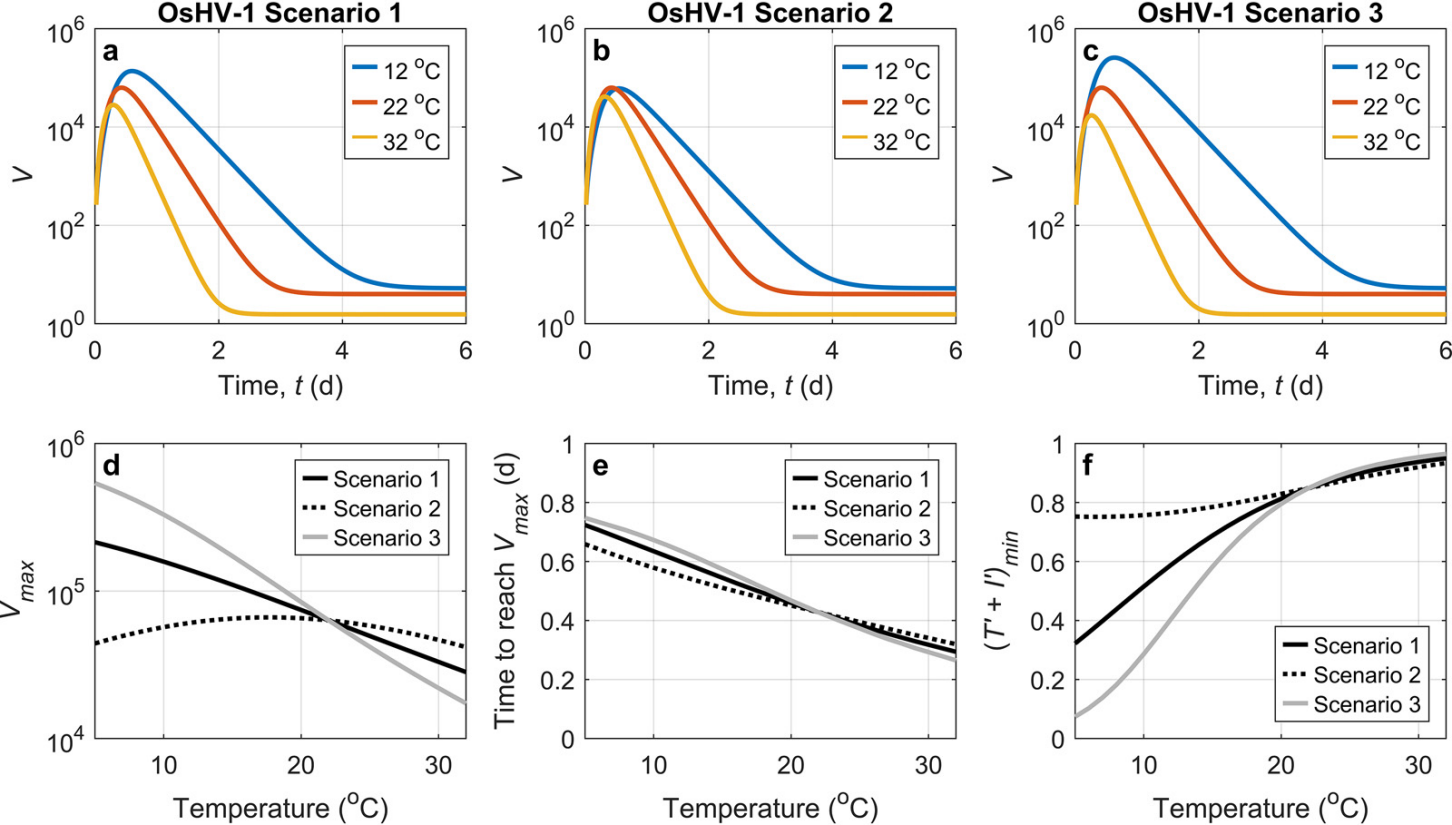
Three OsHV-1 scenarios showing the impact of temperature on viral dynamics in oysters for viruses in group 1. (a to c) Viral dynamics in oysters (viral DNA copies per nanogram of oyster DNA) after infection at temperatures (Temp) of 12°C, 22°C, and 32°C for the three scenarios, respectively. The parameter set at 22°C uses that in Table 1 for the A2 family. (d) Relationships of the maximum virus concentration in the oyster (*V*_max_) with temperature for the three scenarios. (e) Relationships of the time to reach *V*_max_ for the three scenarios. (f) Relationships of the minimum normalized target cells [(*T*′ + *I*′)_min_] to temperature for the three scenarios. The filtration rate with a temperature impact uses the empirical equation for *C. gigas* (34), *f *= 4.825 − 0.013 × (Temp − 18.954)^2^ (in liters per hour), by assuming a total dry weight of 1 g. The effect of temperature on the metabolic rates of oysters is assumed to be equal to the reported effect of temperature on the oxygen consumption rate of oysters (34) that leads to a *Q*_10_ temperature coefficient of 1.5. The net production rate of uninfected target cells (s′_T_net) is assumed to increase with temperature (*Q*_10_ = 1.5). Based on the literature showing the trend of each immune process with increasing temperatures (44), $\text{θ}p_{0}^{\prime }$, *k_p_*, *k*_β_, and *c* are assumed to increase with temperature, with a *Q*_10_ of 1.5, while β_0_ is assumed to be negatively correlated with temperature, with a *Q*_10_ of 0.667 (=1/1.5). In this particular experiment, *c* and (ε*_o_f*)/σ at 22°C are assumed to be 4.2 day^−1^ and 0.3 day^−1^, respectively. The three scenarios for OsHV-1 are conducted for testing different relationships between the parameter δ and temperature that are still unclear because both pro- and antiapoptotic effects by oysters vary with temperature (44). Scenario 1 assumes a constant δ over the range of temperatures, scenario 2 assumes that δ decreases with increasing temperature with a *Q*_10_ of 0.667, and scenario 3 assumes that δ increases with temperature with a *Q*_10_ of 1.5. Other parameters (ε*_i_*, ε*_o_*, σ, *V_l_*, and $I_{0}^{\prime }$) are assumed to be independent of temperature.

The results of the time series for the three scenarios are presented in Fig. 4a to c. Three temperatures (12°C, 22°C, and 32°C) are selected to show that temperature significantly alters the viral dynamics in oysters. The decreasing rate of viral concentration after the peak level becomes greater at higher temperatures due to the increase in the removal rate [(ε_o_/σ)*f* + *c*]. Without considering any changes in the virus concentration in the surrounding water (*V_l_*), the equilibrium viral level decreases with temperature in all three scenarios. It is interesting to examine how temperature impacts the maximum virus concentration in the oyster (*V*_max_) during the acute phase and the time to reach *V*_max_ (Fig. 4d). In scenarios 1 and 3, *V*_max_ is negatively correlated with temperature. In scenario 2, however, *V*_max_ does not show a monotonic correlation with temperature, and the highest value is reached at the middle temperatures. All three scenarios show that the time to reach *V*_max_ is negatively correlated with temperature (Fig. 4e), which is consistent with the laboratory experiment described previously by Petton et al. (6).

The normalized target cell value (*T′* + *I′*) is the ratio of the total target cells in the infected oyster to the total target cells if the oyster has not been infected, and a very low *T′* + *I′* value may result in a low chance of survival during infection. For example, studies show that a high mortality rate of virally infected shrimp occurs when the density of the total hemocytes becomes significantly decreased (64). In the numerical experiment, the minimum normalized target cell value, (*T′* + *I′*)_min_, is generally positively correlated with temperature for all the three scenarios (Fig. 4f), suggesting that a higher temperature may at least contribute to, if it is not the only factor leading to, better conditions for the oyster and increasing chances of survival. The value of *T′* + *I′* is regulated greatly by δ and, thus, apoptosis during infection, as shown by its variations that are expressed by the sum of equations 25 and 26: d(T′+ I′)/dt= s′_T_net−δ*I′*. The relatively large difference in the values of (*T′* + *I′*)_min_ at low temperatures in the three scenarios indicates, from a numerical modeling perspective, that apoptosis may play an important role in regulating viral proliferation in oysters. In addition, among the three scenarios, scenario 2 has the highest δ and the largest values of (*T′* + *I′*)_min_ at low temperatures. This indicates that those oysters having a greater net proapoptotic effect might be better able to survive at low temperatures. Note that a virally induced decrease in the number of target cells may be only one factor contributing to oyster death. For example, as proposed previously by de Lorgeril et al. (12), OsHV-1 infection may lead to subsequent bacterial infection by impairing antibacterial defense. This is suggested to be key in ultimately causing oyster death. Thus, the mortality of the virus may not be correlated monotonically with the number of target cells, and lower temperatures that correspond generally to lower values of (*T′* + *I′*)_min_ may not necessarily decrease the chances of oyster survival. This was demonstrated in a laboratory experiment where juvenile *C. gigas* oysters were exposed to OsHV-1, and maximum mortalities were observed at between 16°C and 22°C, with lower mortality rates above and below that temperature range (6).

Overall, the model results suggest that oysters at higher temperatures seem to be more resistant to viral infection, which agrees with the suggestion based on results from the transcriptomic study described previously by Delisle et al. (44).

### Virus group 2: depuration, bioaccumulation, and long-term equilibrium.

Depuration is a postharvest treatment to reduce contaminant levels in bivalve shellfish (15, 65). This can be done by placing oysters in a tank (*V_l_* = 0) with clean flowthrough water for a relatively short period of time (20) or moving them from contaminated (i.e., restricted or condemned) waters to a clean noncondemned natural location for a specified length of time (66), typically at least 14 days depending on local regulations (67). In cases where *V_l_* is zero, the viral dynamics during depuration are given by solving equation 4:

(6) $$V=V_{0}\exp\left[-\left(\frac{\varepsilon_{o}}{\sigma }f+c\right)t\right]$$

where *V*_0_ is *V* at the beginning. Equation 6 shows that *V* decreases exponentially, consistent with observations and as also suggested by other models (29, 30). The efficacy of depuration depends on the removal rate, (ε*_o_*/σ)*f* + *c*, and hence varies with the in-host elimination rate, *c*; the filtration rate, *f*; and the shedding fraction, ε*_o_*. Thus, any changes leading to a higher value of (ε*_o_*/σ)*f* + *c* can result in a more rapid decrease in virus levels in oyster tissues (e.g., see Fig. 2b). Clearly, the depuration efficacy varies with virus type (Fig. 2a) due to variations among viruses in their responses to antiviral defenses and due to differences in selective accumulation. The removal of NoV, for example, is much less efficient than the removal of many other viruses (15, 18), and therefore, caution needs to be exercised when using one virus type as a surrogate for another virus.

**FIG 5:**
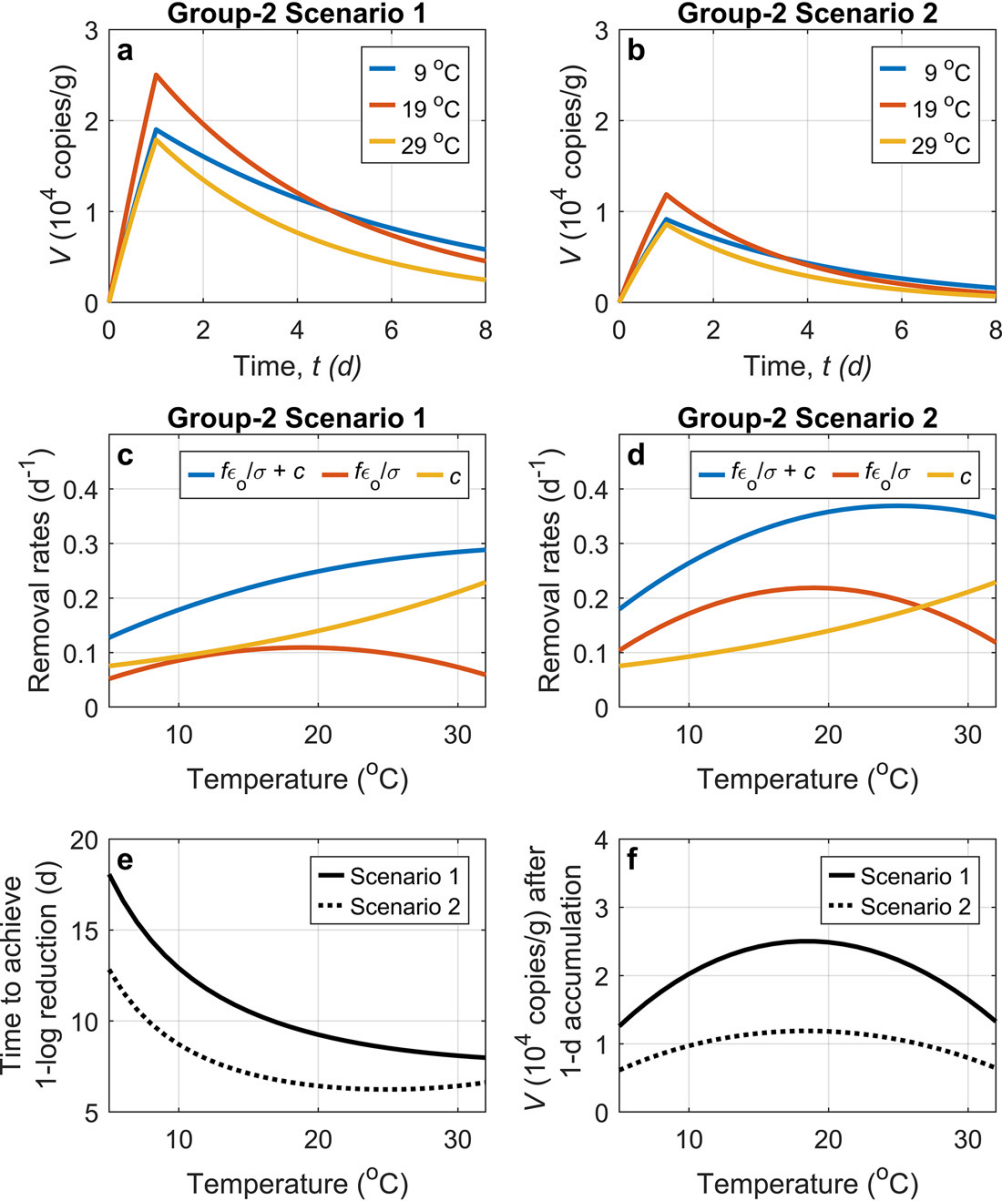
Two model scenarios showing the impacts of temperature on viral dynamics in oysters for viruses in group 2. Both scenarios experience a 1-day bioaccumulation experiment with a constant virus concentration in the surrounding water (*V_l_* = 7.92 × 10^8^ copies/m^3^), followed by a 7-day depuration in clean water (*V_l_* = 0). (a and b) Viral dynamics in oysters during the bioaccumulation experiment and the following depuration at temperatures of 9°C, 19°C, and 29°C for the two scenarios, respectively. (c and d) Relationships of the removal rate (and its two components) with temperature for the two scenarios, respectively. (e) Time to achieve a 1-log reduction of the virus concentration in oysters in response to changes in temperature. (f) Virus concentration in oysters after the 1-day bioaccumulation experiment. The two scenarios differ only in the two parameters related to selective accumulation (ε*_i_* and ε*_o_*). In scenario 1, ε*_i_* is 3.28 × 10^−4^, and ε*_o_*/σ is 1.89. In scenario 2, ε*_i_* is set to be half that in scenario 1, while ε*_o_* is doubled, and this results in an ε*_i_* of 1.64 × 10^−4^ and an ε*_o_*/σ of 3.78. The difference in ε*_i_* and ε*_o_* can be due to different virus species (e.g., NoV versus TV), different strains of the same species (e.g., NoV GI versus GII), or different seasons for one strain (e.g., NoV GI in summer versus winter). The filtration rate with temperature impacts uses the empirical equation for *C. gigas* (34), *f *= 4.825 − 0.013 × (Temp − 18.954)^2^ (in liters per hour), by assuming a total dry weight of 1 g. The units of *f* are converted to meters per day, and *f* is further reduced in half as a prescribed effect of other environmental factors. The response of the in-host clearance/elimination rate (*c*) to temperature is assumed to follow that of the oxygen consumption rate (34) and uses the equation *c *= 0.089 × 1.041^(Temp − 9)^ (equivalent to a *Q*_10_ temperature coefficient of 1.5).

Environmental conditions also affect these parameters and, therefore, the depuration efficacy. Within a certain range, as temperature increases, both *f* and *c* increase, and therefore, the efficacy of depuration increases. This positive relationship with temperature has been supported in many experiments for several different virus types (22, 68). Nevertheless, the relationship of the filtration rate, *f*, to temperature is not monotonic, e.g., *f* reaches a maximum of around 27°C for C. virginica (24) and 19°C for *C. gigas* (34). This indicates that the linear increase in the efficacy may shift to a decrease when the temperature goes over a certain threshold. In a designed experiment using the in-host model (Fig. 5), the depuration efficacy reaches its highest level at 23°C to 27°C for *C. gigas* in scenario 2 when the filter-out/shedding rate (ε*_o_*/σ)*f* contributes substantially to the virus removal rate.

Besides temperature, the model also suggests that the efficacy of depuration can be regulated by other factors that affect the physiological state of the oysters, especially as the filtration rate, *f*, is impacted. An increased *f* enhances efficacy. This may explain the results of experiments where feeding oysters with phytoplankton facilitates a decrease in virus levels in oysters compared to those without feeding (54) since the addition of phytoplankton to sand-filtered water increases TSS. The physiological state of *C. virginica* is heavily influenced by salinity, and thus, salinity can impact both the filtration rate and the clearance rate of this oyster species and, therefore, the ability to retain or clear viruses (19). Thus, it is recommended that depuration be conducted in environments conducive to high filtration rates. Also, as the expression levels of specific ligands for selectively accumulating some viruses (e.g., NoV GI [50]) are low in summer, a higher shedding fraction, ε*_o_*, leading to a higher removal rate is expected for those viruses in warmer months than in winter, and this indicates that oysters harvested in summer may require less time to be depurated (e.g., see Fig. 5e).

The bioaccumulation process in oyster tissues from a health status is described by equation 4 with a zero *V*_0_. Compared to the depuration process, bioaccumulation also depends on the source term, (ε*_i_*/*m*)*fV_l_*. Thus, both the source and sink terms during bioaccumulation are impacted by environmental conditions, which need to be studied on a case-by-case basis. Specifically, for those laboratory experiments with a high viral load for bioaccumulation, the increase of *V* is dominated by (ε*_i_*/*m*)*fV_l_*, and *V* increases with a higher bioaccumulation fraction, ε*_i_*. Also, if laboratory experiments are conducted for examining how the bioaccumulation efficiency varies with temperature, ε*_i_* is expected to be fixed for particular virus and oyster species during the short experimental period, and the bioaccumulation efficiency will be positively correlated with *f* and show a non-monotonic relationship with temperature, as simulated by our model experiment (Fig. 5f). For oysters in nature, while the filtration rate, *f*, may increase with increasing temperature as the season changes from winter to summer, the bioaccumulation fraction, ε*_i_*, may decrease for those viruses showing seasonal variability in bioaccumulation, and the effects of the increased *f* and the decreased ε*_i_* on the accumulation act against each other. The latter may compete over the former for NoV GI and F^+^ coliphage as they are observed to be high in winter but low in summer (21). Our model experiment also shows that with a reduction of ε*_i_* by half, the viral bioaccumulation at 19°C with a higher *f* in scenario 2 is less efficient than that at 9°C in scenario 1 (Fig. 5f), where 19°C and 9°C are close to summer and winter water temperatures reported in a study in France (50). Thus, through parameters ε*_i_* and ε*_o_*, our model mathematically describes that both higher accumulation and lower depuration efficacy contribute to outbreaks in winter months, consistent with previously reported observations (14).

After an extended period of time holding oysters in a relatively stable environment, the virus level will approach an equilibrium value. Analysis of the long-term equilibrium of group 1 viruses also applies to group 2 viruses, with the only difference being that group 2 viruses do not undergo in-host viral production. Whether long-term equilibrium will decrease to zero depends on the viral concentration in the surrounding water, *V_l_*. With depuration in clean water, equilibrium approaches zero. In natural waters or in laboratory experiments where the virus concentration is maintained at a certain level, equilibrium will be above zero. For example, Doré et al. (66) showed that NoV in oysters was maintained at a relatively constant level (1,100 to 2,900 copies/g) over 1 month (9 February to 15 March 2010) in nine samples of oysters from a main harvest area in Ireland, although the virus levels in those samples relayed to clean natural waters nearby and then depurated further in tanks at elevated temperatures demonstrated significant decay. As shown in equation 5, a higher *V_l_* value will lead to a higher equilibrium value of V*.

The dose effect influences the bioaccumulation process and long-term equilibrium. If the virus concentration in the water is fixed, i.e., a constant *V_l_*, the input of virus is unlimited, and a change in *V_l_* changes only the equilibrium value but does not change the time to reach equilibrium. In this case, if all other conditions remain constant, an increase in the filtration rate increases viral uptake from the water and, hence, results in higher equilibrium, V*. However, if the viral dose is fixed, such as in laboratory trials where the virus is added only at the beginning of the experiment, *V_l_* decreases following the initial bioaccumulation. The time to reach equilibrium also depends on *V_l_*. A higher filtration rate decreases the time required to reach equilibrium but does not necessarily increase the equilibrium value.

### Implications of the modeling framework.

This study proposes a model that simulates nonlinear viral dynamics in oysters. This model provides new insight into the viral dynamics in oysters. Our results reveal that many processes contributing to viral dynamics can be impacted by environmental conditions. Overall, the corresponding variation in dynamics is nonlinear, while within a certain range of environmental conditions, the dynamics can exhibit linear properties. For example, for group 1 viruses, the model indicates that oysters at higher temperatures may be more resistant to viral infection. The model also demonstrates that oyster filtration behavior as well as the in-host production of virus and clearance of viruses are essential components of the virus-oyster interaction. Filtration contributes to both viral accumulation and removal in oysters, and it is nonlinear, with temperature, salinity, and TSS substantially affecting the dynamics of the interaction between viruses and oysters. For group 2 viruses, in order to achieve high efficiency, depuration should be conducted in an environment that will promote high filtration rates. Future laboratory experiments are needed that incorporate careful measurements of the effects of variations in temperature, salinity, and TSS on virus accumulation and removal in oysters and that report details from experiments, including oyster weight and size and viral loads in various oyster tissues, to help parametrization in modeling.

Model performance depends on model kinetic parameter values. Some of the model parameters can be directly measured, such as the filtration rate, while others can be calibrated against observations. Uncertainty is expected to exist in the calibrated values of parameters, like those in the example case studies (Table 1), when the data points from each laboratory experiment are limited for calibration. Changes by 2- or 10-fold in the values of some parameters can lead to large differences in the viral dynamics, as shown in the sensitivity test (Fig. 3). These parameters have lower uncertainties than those leading to small differences. For example, the viral dynamics are sensitive to the value of the removal rate, (ε*_o_*/σ)*f* + *c*, which indicates that the estimated removal rate has low uncertainty. The viral dynamics are not sensitive to changes in s′_T_net, indicating that this parameter has high uncertainty; however, the insensitivity also suggests that the uncertainty of this parameter has little impact on the model results or its implications. Overall, uncertainties exist and vary among different parameters. The uncertainty analysis warrants further examination in future studies. As data are expanding as a result of more intensive studies, the accuracy of parameter values can be expected to be improved. In addition, studies with different species and stocks of oysters and different strains of viruses will provide additional information on the host-virus interaction. Different oyster species and stocks have various susceptibilities to different variants of oyster herpesviruses, for example (56, 57, 69). Therefore, parameter values need to be calibrated like all models for different species.

For the purpose of providing a framework for describing viral dynamics in oysters and examining controlling factors, our model adopts the basic model of human virus infection and works well for simulating in-host viral dynamics. Clearly, more experiments are needed to examine how the model parameters vary with different factors for a better representation of the viral dynamics. Also, limitations exist in the current version as some processes are simplified. A more sophisticated model may include an additional latent (or eclipse) phase of infection or explicitly consider the effects of a type I IFN-like immune response on viral replication and spread (31, 70). Nevertheless, due to the limited available data in culture experiments and the uncertainties in parameters, the inclusion of these components often does not show statistically significantly better results in data fitting than the basic model (31). This study focused on developing a model for in-host viral dynamics; we confined our model simulations to within the oyster and considered the viral concentration in water (*V_l_*) an external parameter rather than explicitly simulating it. If an explicit simulation of *V_l_* is needed for a realistic case, it is straightforward to couple the in-host model with other models that simulate *V_l_* explicitly, such as disease transmission/epidemic models (e.g., see references 27 and 28).

### Conclusions.

In this study, we developed a process-based model to provide a modeling framework for fully describing viral dynamics in oysters. The in-host model builds a bridge for better communications between different disciplines studying virus-oyster interactions and theoretically describes complex interactions of various in-host processes and nonlinear viral responses to changes in physiological and environmental conditions. The model can be used to revisit the relationships between viral dynamics and various factors by experiments, predict unknown relationships, and help to propose hypotheses and design experiments. It can also be coupled with population models and transmission models to investigate oyster population dynamics under viral infection and the transmission of viruses between oysters, which will help with better prediction and management of outbreaks. In addition, this modeling framework can be easily adapted for studying other shellfish species such as clams.

## MATERIALS AND METHODS

### Model development.

A mathematical model was developed for providing a framework for studying in-host viral dynamics in oysters. For both virus groups (i.e., group 1 viruses that can infect oyster cells and replicate in oysters and group 2 viruses that do not have adverse effects on the host), filtration is the process through which viruses in the water are transferred to oysters (filter-in) and viruses in oysters are moved out of the oysters to the surrounding water (filter-out or viral shedding). It is assumed that virus particles primarily enter and leave oysters through filtration. A fraction of the filtered-in virus will be accumulated in the oyster tissues, and it is this portion that actually contributes to the viral dynamics in oysters, while the remaining portion is directly removed during filtration. Laboratory experiments suggest that this fraction is generally very small (54). The fraction varies with virus types and other factors, and one reason for this is that oysters have specific ligands that can selectively accumulate certain viruses (16). During filtration, a portion of viruses accumulated in oyster tissues can be filtered out, and the retained fraction depends on selective accumulation and in-host physical trapping that are controlled by factors including virus types and environmental conditions (18). The dynamics of the virus concentration in oysters are also controlled by the various viral clearance strategies, including digestion, inactivation by the innate immune system or diapedesis, pseudofecal rejection, and defecation. For group 1 viruses, replication within the host can be a major source of the virus within the oyster, in addition to the “filter-in” process.

For group 1 viruses, the in-host viral dynamics after initial infection can be described by the widely used basic model of human virus infection (31, 70), with an adaptation for oyster filtration behaviors. The model parameters for the developed model and its transformed forms are listed in Table A1 in the appendix, and the connections between model forms are illustrated in Fig. S1 in the supplemental material. The model has three coupled equations for the dynamics of uninfected target cells, *T*^′^ (cells/oyster); infected cells, *I*^′^ (cells/oyster); and the virus concentration in oyster tissues, *V̂* (virus copies/oyster), respectively:

(7) $$\frac{d\hat{T}}{dt}=-\hat{\beta }\hat{T}\hat{V}-\delta_{T}\hat{T}+{\hat{s}}_{T}$$

(8) $$\frac{d\hat{I}}{dt}=\hat{\beta }\hat{T}\hat{V}-\delta \hat{I}$$

(9) $$\frac{d\hat{V}}{dt}=\hat{p}\hat{I}-\hat{a}\hat{\beta }\hat{T}\hat{V}+\varepsilon_{i}fV_{l}-\frac{\varepsilon_{o}}{\sigma }f\hat{V}-c\hat{V}$$

with an initial condition (*T̂*_0_, *Î*_0_, *V̂*_0_).

The governing equation for the dynamics of group 2 viruses is a specific case of equations 1 to 3, with β̂ =*p̂* = 0. Thus, equations 7 to 9 become decoupled and return a simple bioaccumulation model for the dynamics of *V̂*:

(10) $$\frac{d\hat{V}}{dt}=\varepsilon_{i}fV_{l}-\frac{\varepsilon_{o}}{\sigma }f\hat{V}-c\hat{V}$$

with an initial condition *V̂*_0_.

In practice, the units of virus concentration in oysters may be studied or reported in units other than virus copies per oyster, such as virus copies per gram of tissue. In this case, the units of parameters β and *p* in the model must also be adjusted. We denote *V* the virus concentration in oyster tissues in one reported unit, distinguishing it from *V̂* in units of virus copies per oyster, and also *T* and *I* the uninfected and infected target cells in reported units, respectively. Next, we set θ and ω as the conversion factors to convert the units of copies per oyster and cells per oyster to the new set of units, respectively, i.e., *V*=θ*V̂*, *T*=ω*T̂*, and *I*=ω*Î*. Substituting the expressions *V̂*=*V*/θ, *T̂*=*T*/ω, and *Î*=*I*/ω into the governing equations 7 to 9 returns

(11) $$\frac{dT}{dt}=-\left(\frac{\hat{\beta }}{\theta }\right)TV-\delta_{T}T+\left(\omega {\hat{s}}_{T}\right)$$

(12) $$\frac{dl}{dt}=\left(\frac{\hat{\beta }}{\text{θ}}\right)TV-\text{δ}I$$

(13) $$\frac{dV}{dt}=\text{θ}\left(\frac{\hat{p}}{\text{ω}}\right)I-\text{θ}\left(\frac{\hat{a}}{\text{ω}}\right)\left(\frac{\hat{\beta }}{\text{θ}}\right)TV+{\text{θε}}_{i}fV_{l}-\left(\frac{{\text{ε}}_{o}}{\text{σ}}f+c\right)V$$

It was assumed that θ and ω are constant over the study period. By setting β=β̂/θ, *p*=*p̂*/ω, *a*=*â*/ω, and *s_T_*=ω*ŝ_T_*, equations 11 to 13 become

(14) $$\frac{dT}{dt}=-\text{β}TV-{\text{δ}}_{T}T+s_{T}$$

(15) $$\frac{dl}{dt}=\text{β}TV-\text{δ}I$$

(16) $$\frac{dV}{dt}=\text{θ}pl-\text{θ}a\text{β}TV+{\text{θε}}_{i}fV_{l}-\left(\frac{{\text{ε}}_{o}}{\text{σ}}f+c\right)V$$

with an initial condition (*T*_0_, *I*_0_, *V*_0_) = (ω*T̂*_0_, ω*Î*_0_, θ*V̂*_0_). It can be seen that by doing this, only the conversion factor θ appears explicitly in two terms in equation 16.

Specifically, if the new units are grams per oyster for *T* and *I* and virus copies per gram for *V*, we have θ = *m*^–1^ (oysters per gram), where *m* (grams per oyster) is the weight of the total target cells in one oyster if the oyster is not infected and ω (grams per cell) denotes the weight of one oyster cell. β, *p*, α, and *s_T_* correspondingly have the units of grams per copy per day, copies per gram per day, copies per gram, and grams per oyster per day, respectively. Equation 16 can be rewritten as

(17) $$\frac{dV}{dt}=\frac{pl}{m}-\frac{a}{m}\text{β}TV+\frac{{\text{ε}}_{i}}{m}fV_{l}-\left(\frac{{\text{ε}}_{o}}{\text{σ}}f+c\right)V$$

The equation for group 2 viruses becomes

(18) $$\frac{dV}{dt}=\frac{{\text{ε}}_{i}}{m}fV_{l}-\left(\frac{{\text{ε}}_{o}}{\text{σ}}f+c\right)V$$

Equations 14, 15, 17, and 18 are used as the basic form of the in-host model (equations 1 to 3) in this study.

### Equilibrium and long-term effect.

For group 1 viruses, the virus level in oysters approaches an equilibrium in surviving oysters, and it can be solved by the steady-state solution of equation 17, i.e., *dT*/*dt* = *dI*/*dt* = *dV*/*dt* = 0. The steady-state solution for *V* reads

(19) $$V^{*}=\frac{q+\frac{{\text{ε}}_{i}}{m}fV_{l}}{\frac{{\text{ε}}_{o}}{\text{σ}}f+c}$$

where *q* = (*p*/*m*/δ − *a*/*m*)(*s_T_* − δ_T_T*) and T* is the steady-state solution for *T*. The values of all other parameters in equation 19 are also their long-term mean values. Besides the nontrivial equilibrium, a trivial equilibrium (V* = 0) also exists if *V_l_* is zero.

The long-term accumulation of group 2 viruses can be estimated by solving equation 18 under steady state (*dV*/*dt* = 0), which reads

(20) $$V^{*}=\frac{\frac{{\text{ε}}_{i}}{m}fV_{l}}{\frac{{\text{ε}}_{o}}{\text{σ}}f+c}$$

The stabilities of the long-term equilibriums for group 1 and group 2 viruses are analyzed in the supplemental material.

### Normalized equations for simulating laboratory experiments.

The parameters in the model can be calibrated against observational data from laboratory experiments. To better use the in-host model, it may be helpful to normalize *T* and *I*, which are often unreported in observations. For *T* and *I* in any given units, by setting *T*′ = *T*/*T_a_*, and *I*′ = *I*/*T_a_*, where *T_a_* is the initial number of total target cells if the oyster is not infected, having the same units as *T*. From equation 14, we get *T_a_* = *s_T_*/δ*_T_* when *V* is zero. Using the same procedure to obtain equations 11 to 13, we can transform equations 14 to 16 into

(21) $$\frac{dT^{\prime }}{dt}=-\text{β}T^{\prime }V-{\text{δ}}_{T}T^{\prime }+s\prime {\;}_{T}$$

(22) $$\frac{dI^{\prime }}{dt}=\text{β}T^{\prime }V-\text{δ}I^{\prime }$$

(23) $$\frac{dV}{dt}=\text{θ}p^{\prime }I^{\prime }-\text{θ}a^{\prime }\text{β}T^{\prime }V+{\text{θε}}_{i}fV_{l}-\left(\frac{{\text{ε}}_{o}}{\text{σ}}f+c\right)V$$

with initial conditions ($T_{0}^{\prime }$, $I_{0}^{\prime }$, *V*_0_), where *p′*=*pT_a_*=*p̂*(*T_a_*/ω), *a′*=*aT_a_*=*â*(*T_a_*/ω), and *s′* *T*=*s′* _*T*_/*T_a_*=(ω/*T_a_*)*ŝ_T_*. Correspondingly, the long-term equilibrium of *V* is

(24) $$V^{*}=\frac{q^{\prime }+{\text{θε}}_{i}fV_{l}}{\frac{{\text{ε}}_{o}}{\text{σ}}f+c}$$

where $q^{\prime }=(\theta p^{\prime }/\delta -\text{θ}a^{\prime })(s\prime {\;}_{T}-\delta_{T}{\mathrm{T\prime}}^{*})$ and *q*′ = *q* in equation 19 if θ = *m^−^*^1^.

For group 1 viruses, in the acute infection phase, the dynamics of the virus concentration in oysters is regulated by dominant processes. To reduce the uncertainties in calibrating parameters that do not have critical effects on the viral dynamics, we can reduce the number of parameters in the model by retaining only the dominant processes when simulating the acute phase. It is reasonable to assume that the death rate of an uninfected target cell, δ*_T_*, is sufficiently low, and it can be neglected in the calculation by accounting for the loss implicitly in s′_T_net, i.e., $s\prime {\;}_{T}\text{net}=s\prime {\;}_{T}-{\text{δ}}_{T}T^{\prime }$. s′_T_net can be considered the net production of uninfected target cells due only to mechanisms 2 and 3 listed in Results, which is assumed to be constant during the short period of the acute phase. The loss term (−θ*a*′β*T*′*V*) may also be considered small enough to be neglected during the acute phase. Thus, we have

(25) $$\frac{dT^{\prime }}{dt}=-\text{β}T^{\prime }V+s\prime {\;}_{T}\text{net}$$

(26) $$\frac{dI^{\prime }}{dt}=\text{β}T^{\prime }V-\text{δ}I^{\prime }$$

(27) $$\frac{dV}{dt}=\left(\theta p^{\prime }\right)I^{\prime }+{\theta \varepsilon }_{i}fV_{l}-\left(\frac{{\text{ε}}_{o}}{\text{σ}}f+c\right)V$$

with initial conditions ($T_{0}^{\prime }$, $I_{0}^{\prime }$, *V*_0_). For experiments starting from low *V*_0_ and $I_{0}^{\prime }$, it may be assumed that $T_{0}^{\prime }=1-I_{0}^{\prime }$.

For the acute infection phase in the experiments, a type I IFN-like immune response can cause decreases in viral replication and spread (70). Without losing generality, this decrease can be considered by assuming exponential decreases in *p* and β in the forms $p=p_{0}e^{-k_{p}t}$ and $\text{β}={\text{β}}_{0}e^{-k_{\text{β}}t}$, where *p*_0_ and β_0_ denote the viral replication rate and the infection rate at the beginning, respectively; *k_p_* and *k*_β_ have units of per day. For the parameter θ*p*′ in equation 27, we have $\text{θ}p^{\prime }=\text{θ}p_{0}^{\prime }e^{-k_{p}t}$, where $p_{0}^{\prime }=p_{0}T_{a}$. Other parameters may be assumed to be constant during the laboratory experiments. Thus, in addition to calibrating $I_{0}^{\prime }$, we need to calibrate the following parameters based on observational data:$\theta p_{0}^{\prime }$, β_0_, *k_p_*, *k*_β_, δ, s′_T_net, θε*_i_fV_l_*, and (ε*_o_*/σ)*f* + *c.* Note that the filter-in (θε*_i_fV_l_*) is comparably smaller than in-host viral production, and its role becomes significant only after the acute phase; thus, for the purpose of fitting, θε*_i_fV_l_* can be simply set constant at the long-term equilibrium value without causing large biases.

### Example case studies.

Data sets of experimental results of the in-host dynamics of OsHV-1 in two oyster families (39) and the in-host dynamics of NoV and TV (54) were fitted using the model for group 1 and group 2 viruses, respectively. The mean concentration of OsHV-1 reported previously by Segarra et al. (39) was reported in units of viral DNA copies per nanogram of total DNA, which was converted to units of viral DNA copies per nanogram of oyster DNA by calculating how much of the total DNA was viral DNA. The genome size of OsHV-1 is about 200,000 bp to 207,000 bp depending on the OsHV variant; therefore, the weight is about 0.204 to 0.212 fg/viral genome. The genome size of OsHV-1 used in the experiment is 207,439 bp (10), so we used 0.212 fg/viral genome to convert the units with the following expression: $V_{\text{OsHV}}=V_{\text{OsHV}}^{\text{rep}}\text{/}(1–V_{\text{OsHV}}^{\text{rep}}\times 0.208\times 10^{-6})$, where $V_{\text{OsHV}}^{\text{rep}}$ and *V*_OsHV_ are the reported and converted OsHV-1 concentrations, respectively. The concentrations of NoV and TV reported previously by Drouaz et al. (54) were reported in units of viral RNA copies per gram of digestive tissue (DT). Equations 25 to 27 were used for fitting OsHV-1 experimental data, and equation 18 was used for fitting NoV and TV data. For the OsHV-1 experiments, the nonlinear model was calibrated by trial and error. Briefly, beginning with initial values for the parameters that are assigned based on typical values used for human virus infection as reviewed previously by Ciupe and Heffernan (70), we assigned a series of values for each parameter and looked for the set of values that led to the best match of model results against the experimental results. The coefficient of determination, *r*^2^, and the root mean squared error were used for statistically evaluating each model result. For the NoV and TV simulations of the depuration experiments, the experiments used constantly circulating clean seawater (54); thus, the virus concentration in the surrounding water, *V_l_*, was set to be zero, and calibration was conducted using the linear regression between the log of *V* and the time.

We also calculated the bioaccumulation abilities of NoV and TV in the bioaccumulation experiments reported by Drouaz et al. (54). By assuming that *V *= 0 at the beginning, *t *= 0, we can solve equation 18:

(28) $$V=\left(\frac{\frac{{\text{ε}}_{i}}{m}fV_{l}}{\frac{{\text{ε}}_{o}}{\text{σ}}f+c}\right)\left[1-e^{-\left(\frac{{\text{ε}}_{o}}{\text{σ}}f+c\right)t}\right]$$

From equation 28, the mean bioaccumulation fraction (ε*_i_*) over an experimental period can be computed with *V* at the end of the period and the known values of *V_l_*, *f*, *m*, and the removal rate, (ε*_o_*/σ)*f* + *c*. We computed ε*_i_* for NoV and TV in the DT of oysters over the first hour of the bioaccumulation experiments, where *V* values at 1 h are 537 and 176 viral RNA copies/g DT for NoV and TV, respectively (54). Note that ε*_i_* for the DT is different from that of other tissues. Given the high initial virus concentrations in the water (*V_l_* = 7.92 × 10^8^ viral RNA copies/m^3^ for NoV, and *V_l_* = 1.58 × 10^10^ viral RNA copies/m^3^ for TV), during the short initial period, the decrease in *V_l_* can be neglected, and *V_l_* can be assumed to be constant in the computation. As we focused on the DT, *m* is the mean DT weight of an individual oyster, equal to 0.53 g DT oyster^−1^. The removal rates, (ε*_o_*/σ)*f* + *c*, were computed based on the 8-day depuration experiments following the bioaccumulation experiments under similar conditions. In fact, given the small values of the removal rates (on the order of 0.1 day^−1^) and the short period considered (1 h), the effect of the removal rate on the bioaccumulation of *V* is negligible. This is shown by the fact that

(29) $$\left[1-e^{-\left(\frac{\varepsilon_{o}}{\sigma }f+c\right)t}\right]\approx \left(\frac{{\text{ε}}_{o}}{\text{σ}}f+c\right)t\text{ for}\left(\frac{{\text{ε}}_{o}}{\text{σ}}f+c\right)t\ll 1$$

and equation 28 can be simplified to

(30) $$V\approx \frac{{\text{ε}}_{i}}{m}fV_{l}t$$

This also indicates that the bias in the estimation of ε*_i_* due to the uncertainty of the removal rate is limited by using equation 28 as long as the experimental period is short. The filtration rate, *f*, was computed using an empirical equation for the filtration rate of *C. gigas* (34) at experimental temperatures of 8°C to 10°C. It is expected that TSS also affects *f* of *C. gigas*, in comparison to *C. virginica*, for which a low TSS can reduce its *f* to 0.1× *f* (24). Our calculation implies that the TSS effect reduces *f* at least in half in the bioaccumulation experiment reported by Drouaz et al. (54) by comparing the different removal rates for their 8-day depuration experiments with and without adding phytoplankton. Thus, we assumed that TSS further reduces the filtration rate to 0.1 to 0.5 of its value. This leads to a possible range of *f* values from 0.0089 to 0.044 m^3^ day^−1^ oyster^−1^ for the bioaccumulation experiments.

### Data availability.

The MATLAB code and data used during the present study are available in figshare at https://doi.org/10.6084/m9.figshare.19294580.v1.

## APPENDIX

The model parameters for the developed in-host model and its transformed forms are listed in Table A1.

**TABLE A1:** Notation

Symbol	Description	Unit(s) of measure^*a*^
Parameters in the original model (equations 7–9)		
* t*	Time	Days
*T̂*	Quantity of uninfected target cells	Cells/oyster
*Î*	Quantity of infected cells	Cells/oyster
*V̂*	Virus concn in oyster tissues	Copies/oyster
*T̂*_0_	Initial quantity of uninfected target cells at *t *= 0	Cells/oyster
*Î*_0_	Initial quantity of infected cells at *t *= 0	Cells/oyster
*V̂*_0_	Initial virus concn in oyster tissues at *t *= 0	Copies/oyster
*p̂*	Rate of virus replication in the infected cells	Copies/cell/day
β̂	Infection rate between oyster cells	Oysters/copy/day
*ŝ_T_*	Production rate of new uninfected target cells	Cells/oyster/day
*â*	Copies of virus that are needed to infect 1 target cell	Copies/cell
* *δ	Death rate of infected target cells	Day^−1^
* *δ*_T_*	Death rate of uninfected target cells	Day^−1^
* c*	In-host virus clearance/elimination rate	Day^−1^
* f*	Water filtration rate by an oyster	m^3^/day/oyster
* V_l_*	Virus concn in the surrounding water	Copies/m^3^
* *ε*_i_*	Bioaccumulation fraction, the fraction of virus that binds the tissues after being filtered into the oyster	Unitless
* *ε*_o_*	Shedding fraction, the fraction of virus that cannot be retained in oyster tissues during filter-out/shedding processes	Unitless
* *σ	Vol of individual oyster	m^3^/oyster

Additional parameters in the transformed model (equations 1–3 and 14–18)		
* m*	wt of the total target cells in 1 oyster if the oyster is not infected	g/oyster
* T*	Quantity of uninfected target cells	Case specific; g/oyster in equations 1–3
* I*	Quantity of infected cells	Case specific; g/oyster in equations 1–3
* V*	Virus concn in oyster tissues	Case specific; copies/g in equations 1–3; viral DNA copies/ng of oyster DNA for OsHV-1 and viral RNA copies/g of DT for NoV and TV in the example case studies
* T*_0_	Initial quantity of uninfected target cells at *t *= 0	Case specific; g/oyster in equations 1–3
* I*_0_	Initial quantity of infected cells at *t *= 0	Case specific; g/oyster in equations 1–3
* V*_0_	Initial virus concn in oyster tissues at *t *= 0	Case specific; copies/g in equations 1–3; viral DNA copies/ng of oyster DNA for OsHV-1 and viral RNA copies/g of DT for NoV and TV in the example case studies
* p*	Rate of virus replication per infected cell	Case specific; copies/g/day in equations 1–3
* *β	Infection rate between oyster cells	Case specific; g/copy/day in equations 1–3; ng of oyster DNA/viral DNA copy/day for OsHV-1 in the example case studies
* s_T_*	Production rate of new uninfected target cells	Case specific; g/oyster/day in equations 1–3
* a*	Copies of viruses that are needed to infect 1 quantity of target cells	Case specific; copies/g in equations 1–3
* V**	Steady-state solution for *V*	Case specific; copies/g in equation 5
* T**	Steady-state solution for *T*	Case specific; g/oyster in equation 5
* q*	Steady-state net in-host viral production	Case specific; copies/g/day in equation 5
* *θ	Conversion factor to convert units of *V̂* to new units of *V*, i.e., *V*=θ*V̂*	Case specific; (viral DNA copies/ng of oyster DNA)/(copies/oyster) for OsHV-1 and (viral RNA copies/g of DT)/(copies/oyster) for NoV and TV in the example case studies
* *ω	Conversion factor to convert units of *T̂* and *Î* to new units of *T* and *I*, i.e., *T*=ω*T̂* and *I*=ω*Î*	Case specific

Additional parameters in the normalized model (equations 21–23)		
* T_a_*	Initial quantity of total target cells if the oyster is not infected	Case specific; same as units of *T*
* T*′	Normalized uninfected target cells, *T*′ = *T*/*T_a_*	Unitless
* I*′	Normalized infected target cells, *I*′ = *I*/*T_a_*	Unitless
$T_{0}^{\prime }$	Normalized initial uninfected target cells at *t *= 0	Unitless
$I_{0}^{\prime }$	Normalized initial infected target cells at *t *= 0	Unitless
* p*′	Rate of virus replication in infected cells, *p*′ = *pT_a_*	Copies/oyster/day
s′_T_	Production rate of new uninfected target cells, s′_T_ $=s_{T}\text{/}T_{a}$	Day^−1^
* a*′	Copies of viruses that are needed to infect all target cells in 1 oyster at *t *= 0, *a*′ = *aT_a_*	Copies/oyster
* T*′*	Steady-state solution for *T*′, *T*′* = *T**/*T_a_*	Unitless
* q*′	Steady-state net in-host viral production	Case specific; same as units of *q*

Additional parameters in the normalized model for fitting lab data (equations 25–27)		
s′_T_net	Net production of uninfected target cells, s′_T_net =s′_T_ −${\text{δ}}_{T}T^{'}$	Day^−1^
$p_{0}^{\prime }$	Initial rate of virus replication in the infected cells at *t *= 0, $p_{0}^{\prime }=p_{0}T_{a}$	Copies/oyster/day
* *β_0_	Initial infection rate between oyster cells at *t *= 0	Case specific; ng of oyster DNA/viral DNA copy/day for OsHV-1 in the example case studies
* k_p_*	Decay rate for viral replication rate	Day^−1^
* k*_β_	Decay rate for infection rate	Day^−1^

a “Case specific” indicates that there are no fixed units for that parameter, and the choice of units depends on the specific study.
