# Supplementary material for: A Deterministic Model for Understanding Nonlinear Viral Dynamics in Oysters
Source: Appl Environ Microbiol. 2022 Mar 29;88(8):e02360-21. doi: 10.1128/aem.02360-21 (PMC9046769; doi:10.1128/aem.02360-21)
Supplement: Supplemental file 1 — Section S1 and Fig. S1. Download aem.02360-21-s0001.pdf, PDF file, 0.2 MB [file aem.02360-21-s0001.pdf]

Supplemental Material for

A deterministic model for understanding nonlinear viral dynamics in oysters

Qubin Qin[a#], Jian Shen[a], Kimberly S. Reece[a]

[a]Virginia Institute of Marine Science, William & Mary

Gloucester Point, VA 23062, USA

The supplemental material includes:

Section S1: Stability analysis of the long-term equilibrium.

Figure S1: A list of the in-host model and its transformed forms in this study.

References for supplemental material

S1: Stability analysis of the long-term equilibrium

For Group 2 viruses, it can be easily seen that the equilibrium is stable using the linear stability analysis. Setting $\dot{V} = \frac{dV}{dt}$, it is shown that $\frac{d\dot{V}}{dV} = -\left(\frac{\varepsilon_o}{\sigma} f + c\right)$, which is always below zero, i.e., $\frac{d\dot{V}}{dV} < 0$. This suggests that any small perturbation from the equilibrium V^*, $(V - V^*)$, decays with time so that $V^* = \frac{\frac{\varepsilon_i}{m} f V_l}{\frac{\varepsilon_o}{\sigma} f + c}$ (equation 20) is always stable, and V with any initial condition will approach this equilibrium.

For Group 1 viruses, studies on the stability have been conducted for viral dynamics in a closed system. Our model collapses to a closed system if there are no oyster filtration behaviors that exchange viruses between the oyster inner environment and the surrounding water (i.e., $\frac{\varepsilon_i}{m} f V_l = \frac{\varepsilon_o}{\sigma} f V = 0$). For a closed system, it has been suggested that whether the equilibrium V^* is stable depends on the basic reproduction number R_0 (e.g., 1-3). If $R_0 > 1$, V^* is positive and asymptotically stable (i.e., leading to chronic infections); If $R_0 < 1$, there is no positive V^* and V decreases to zero (i.e., virus is totally removed). When a can be neglected, the expression of R_0 for equations 1-3 is (4):

$$R_0 = \frac{p\beta s_T}{mc\delta\delta_T} = \frac{p\beta T_a}{mc\delta}$$

When the filtration behaviors are included, the system becomes an open system and it is more complicated to analyze the stability of the long-term equilibrium V^* in equation 5 or 19.

It is expected that if the filter-in virus is much less than the in-host production during the long period after the acute infection phase, the dynamics of the open system may be similar to

that of the closed system, and we may neglect the term $\frac{\varepsilon_i}{m} f V_l$. In this case, the equilibrium V^* is close to

$$V^* \approx \frac{q}{\frac{\varepsilon_o}{\sigma} f + c}, \text{ as } q \gg \frac{\varepsilon_i}{m} f V_l.$$

In this case, the stability analysis for the open system with weak exchange resembles that for the closed system, and there exists a R_0 to determine whether V^* is stable. If a is further neglected, then R_0 is close to that for the closed system shown above. If $R_0 > 1$, V approaches V^* that is asymptotically stable. If $R_0 < 1$, oyster has the ability to remove all of the in-host viruses, however, due to the existence of the filter-in process, V remains low but cannot become vanished unless $V_l = 0$.

If the filter-in is the dominant process for increasing the in-host viral concentrations after the acute infection phase, then the dynamics of Group 1 viruses after the acute phase is similar to that of Group 2 viruses and the equilibrium V^* is close to

$$V^* \approx \frac{\frac{\varepsilon_i}{m} f V_l}{\frac{\varepsilon_o}{\sigma} f + c}, \text{ as } q \ll \frac{\varepsilon_i}{m} f V_l.$$

This indicates the equilibrium V^* for Group 1 viruses in this case is positive and stable.

More studies are needed for evaluating the stability of the long-term equilibrium for Group 1 viruses, especially for the conditions that filter-in and in-host production are both important after the acute phase.

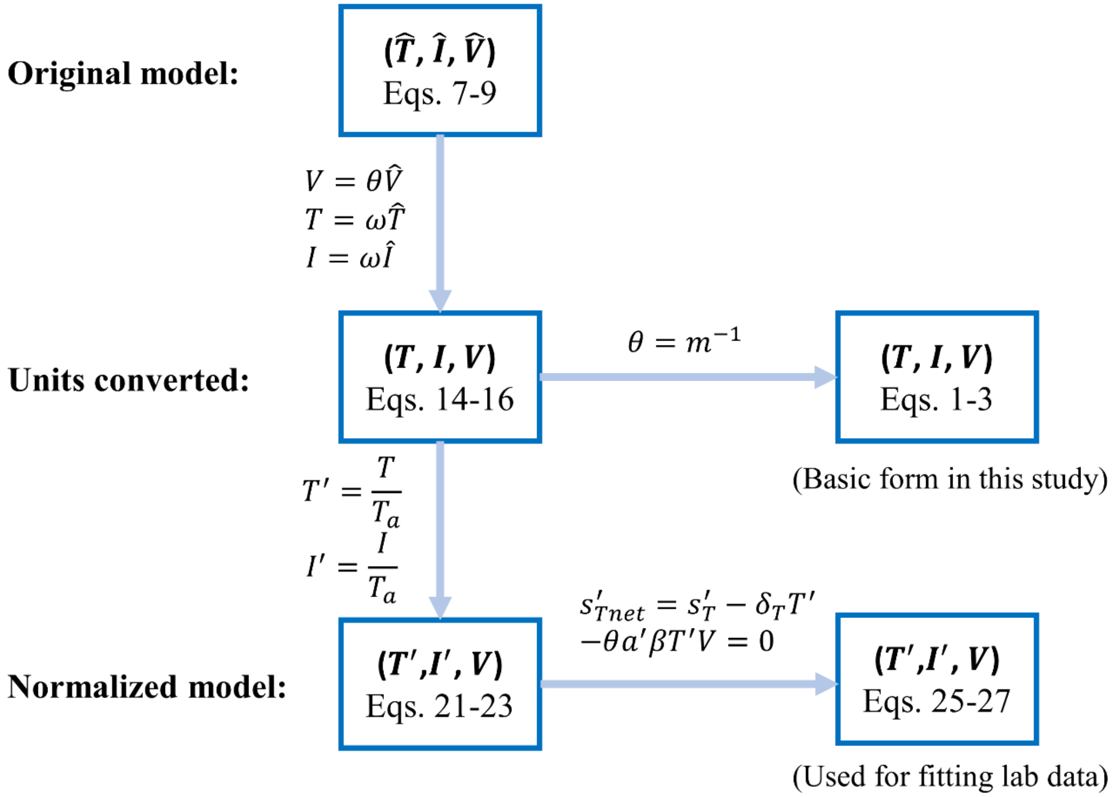

Fig S1. The in-host model and its transformed forms in this study. $\hat{T}$, $\hat{I}$, and $\hat{V}$ have the units of cells/oyster, cells/oyster, and virus copies/oyster, respectively. θ and ω the conversion factors to convert the units of copies/oyster and cells/oyster to the targeted units, respectively. m is the weight of the total target cells in one oyster and has the units of g/oyster. T_a is the total target cells if the oyster is not infected, and T' and I' (unitless) are normalized T and I, respectively.

References:

1. van den Driessche P, Watmough J. 2002. Reproduction numbers and sub-threshold endemic equilibria for compartmental models of disease transmission. Mathematical Biosciences, 180:29e48.

2. Smith HL, De Leenheer P. 2003. Virus dynamics: A global analysis. SIAM Journal on Applied Mathematics, 63(4):1313-1327

3. Korobeinikov A. 2004. Global properties of basic virus dynamics models. Bulletin of Mathematical Biology, 66(4):879-883.

4. Ciupe SM, Heffernan JM. 2017. In-host modeling. Infect Dis Model 2(2):188-202.
